# Phytotherapy of nephrotoxicity-induced by cancer drugs: an updated review

**DOI:** 10.15171/jnp.2017.41

**Published:** 2017-02-16

**Authors:** Saeid Heidari-Soreshjani, Majid Asadi-Samani, Qian Yang, Ali Saeedi-Boroujeni

**Affiliations:** ^1^Medical Plants Research Center, Shahrekord University of Medical Sciences, Shahrekord, Iran; ^2^Student Research Committee, Shahrekord University of Medical Sciences, Shahrekord, Iran; ^3^Institute of Pharmacy and Molecular Biotechnology, University of Heidelberg, Heidelberg, Germany; ^4^Department of Immunology, School of Medicine, Jundishapur University of Medical Sciences, Ahvaz, Iran

**Keywords:** Nephrotoxicity, Cisplatin, Cyclophosphamide, Chemotherapy, Natural compounds

## Abstract

**Context:**

Kidney is one of the vital organs maintaining homeostasis of body and thus dysfunction of kidney affects quality of life and health severely. Anticancer drugs, particularly chemotherapeutic agents, cause high toxicity leading kidney dysfunction and irreparable kidney injury. Therefore, attention has recently been paid to seeking out alternatives such as nature-based drugs that are effective but less toxic. In this regard, this systematic review article is to report and introduce the most important medicinal plants and their derivatives that are used to reduce anticancer drug-induced nephrotoxicity. Evidence Acquisitions: The word nephrotoxicity alongside the words cancer or chemotherapy in combination with some herbal terms such as medicinal plant, plants, herbs, and extracts were administered to search for relevant publications indexed in PubMed.

**Results:**

According to this study, 16 medicinal plants, 12 plant-based derivatives, and three traditional plant-based formulations were found to help control and modulate anticancer drug-induced nephrotoxicity indices.

**Conclusions:**

Anticancer drugs cause nephrotoxicity through activating pathways of oxidative stress, damage-associated molecular patterns (DAMPs) production, inflammatory processes, and cell apoptosis, while medicinal plants and their derivatives can cause reduction in nephrotoxicity and anticancer drugs side effects via their antioxidant and anti-inflammatory properties.

**Implication for health policy/practice/research/medical education:**

Nephrotoxicity is one of the most common side effects induced by anticancer drugs and severely affects the patients’ lives. It plays a significant role in the process of cancer treatment. The findings of this review article will provide researchers and pharmacists with keys to identification of new medicinal plants to modulate and reduce nephrotoxicity and therefore explain routes to accelerate use of natural product drugs in healthcare system.

## 1. Context

Cancer is one of the most important causes of death worldwide such that around 14 million new cases of cancer and 8.2 million deaths due to cancer were reported in 2012. Therefore, early diagnosis and appropriate treatment are considered to be among the most important measures to reduce mortality among cancer patients (1). Although many chemotherapeutic agents are currently available to treat a variety of cancers, side effects due to these drugs have brought about certain limitations facing use of them and eradication of cancer. For example, anticancer drugs are associated with nephrotoxicity, i.e. one of the most common anticancer drug-induced side effects, which can cause temporary or permanent kidney injury and even endanger the lives of cancer patients and therefore intensify their conditions (2-4). Studies have shown that nephrotoxicity is the cause of acute renal injuries in 60% of all clinical cases, and is induced mainly by anticancer drugs and associated with several side effects and mortality (2,5). Nephrotoxicity can be diagnosed through simple blood tests. Assessment of nephrotoxicity through blood tests includes the measurements of blood urea nitrogen (BUN), concentration of serum creatinine, glomerular filtration rate and creatinine clearance.

Anticancer drugs, including cisplatin, oxaliplatin, bleomycin, ifosfamide, carboplatin, mitomycin C, bendamustine, melphalan, vinca, nitrosoureas, methotrexate, capecitabine, irinotecan, gemcitabine and pemetrexed (6-10), play roles in induction of nephrotoxicity. Administration of natural products such as medicinal plants is one of the effective approaches to reduce the complications of various diseases with fewer side effects (11-17). They can be used to reduce anticancer drug-induced side effects because of the good safety and tolerability profile of natural compounds in human (18-21). In this review we report the progress made in utilizing medicinal plants in protecting anticancer drug-induced nephrotoxicity and discuss their mechanisms of action.

## 2. Evidence Acquisition

To retrieve relevant articles to this systematic review, EndNote software was used. Moreover, the word *nephrotoxicity* alongside the words *cancer* or *chemotherapy* in combination with some herbal terms such as medicinal plant, plant, herb and extracts were administered to search for relevant publications indexed in PubMed as the most important medical database.

By means of different combinations of the above search terms, 162 articles on nephrotoxicity, cancer, and chemotherapy that were related to medicinal plants were retrieved while 81 of which were duplicate publications. After excluding duplicate publications, 81 articles were identified by a fully specialized search and their abstracts analyzed. Among these articles, only those that mainly investigated the effects of the medicinal plants and their derivatives in reducing anticancer drug-induced nephrotoxicity were selected and the data needed for this systematic review article were drawn.

## 3. Results

### 3.1. Phytotherapy for anticancer drug-induced nephrotoxicity

#### 3.1.1 Herbal extracts

Pomegranate:******Pomegranate has antioxidant, anti-inflammatory and antimicrobial effects. It is reported that different parts of pomegranate can prevent cisplatin-induced nephrotoxic side effects. Pomegranate seed extract causes decrease in lipid peroxidation and increase in glutathione S-transferase level and activities of superoxide dismutase, glutathione peroxidase, and glutathione S-transferase. Pomegranate seed extract could prevent nephrotoxicity side effects in laboratory rats due to antioxidant and antiapoptotic properties (22). A study demonstrated that pomegranate seed essential oil reduced glucose, protein, serum urate, renal malondialdehyde (MDA), and serum creatinine and increased thiol levels (23). Karwasra et al studied the effects of pomegranate rind extract in induction of nephrotoxicity and renal injuries. The administration of pomegranate rind extract significantly ameliorated increased serum creatinine and BUN, which were induced by cisplatin. In addition, pomegranate exhibited antiapoptotic activity due to the decreased activity of caspase-3 expression (24).

Administration with pomegranate flower extract accompanied by cisplatin has been reported to reduce cisplatin renal toxicity. Moreover, if administered with cisplatin alone, this extract can decrease blood nitrite and tissue damage to renal glomeruli in rats (25). However, Jilanchi et al reported that pomegranate flower extract aggravated renal injuries induced by cisplatin in rats (26). Also, Nasri and Rafieian-Kopaei (27) and El-Arabey (28) have argued that pomegranate flower extract does not seem to be effective as an antioxidant agent in reducing cisplatin nephrotoxicity in female rats, which may be due to the pro-oxidant activity or use of low doses of this extract in the studies. Besides, due to containing phytoestrogens and having estrogenic property, pomegranate flower causes increase in cisplatin nephrotoxicity. Therefore, the effects of pomegranate seeds have been considered mainly in reducing cisplatin-induced nephrotoxic side effects as these seeds are frequently used in traditional medicine and no side effects induced by pomegranate seeds have yet been reported.

Garlic:******A study to investigate the protective effect of garlic (*Allium sativum*) on methotrexate-induced nephrotoxicity in rats demonstrated that aqueous *A*. *sativum* extract caused increase in filtration, improved renal function and activities of antioxidant enzymes. Moreover, garlic extract decreases oxidative stress and prevents morphological changes in the kidney (5). Because of exerting antioxidant, antiapoptotic, and anti-inflammatory effects, garlic decreases MDA level and improves kidney action. In addition, garlic extract protects cisplatin-induced side effects including histomorphological, ultrastructural, and biochemical changes (29).

Green tea: Green tea (*Camellia sinensis*) is a frequently used plant in traditional medicine. Studies on the protective effects of green tea against cisplatin nephrotoxicity in male rat showed that green tea can not only reduce cisplatin destructive changes, but also is effective on increased Pi transport activity (30). Compared with administration of chemotherapy, administration of green tea and royal jelly demonstrated decreased BUN and serum creatinine accompanied with increased glutathione level and MDA production in mice (31).

Grape: Grape contains large amounts of antioxidant phytochemicals such polyphenols, reseveratrol and proanthocyanidins. A study on renal protective effects of grape juice (with skin and seed) showed that no significant change was observed in oxidative stress and antioxidant status in the kidneys of cisplatin-treated mice. However, tubular cell vacuolization, tubular dilatation, and cast formation in kidney tubules were slightly improved after pretreatment with whole grape juice (32). Therefore, given the inconsistent findings of the studies and exerting certain beneficial effects, grape should be further studied for its effects on the protection against anticancer drug-induced renal toxicity.

*Curcuma caesia* Roxb.:* C. caesia* Roxb. is traditionally used in treating various ailments and metabolic disorders in Indian system of medicine. The protective effects of *C. caesia* Roxb*.* against chemotherapeutic drug cyclophosphamide was evaluated. The study showed that cyclophosphamide exerted toxicity on the kidney and liver in mice, and use of methanolic *C*. *caesia* Roxb extract reduced serum levels of aspartate aminotransferase (AST) and alanine transaminase (ALT) and kidney peroxidation in the treatment group (33).

*Rubia cordifolia: R. cordifolia*, which is commonly used in Indian traditional medicine, is from family Rubiaceae and has antioxidant properties. A study on *R*. *cordifolia* extract effects in cisplatin-treated mice demonstrated that this plant reduced serum creatinine and urea levels as well as lipids peroxidation, and therefore could modulate nephrotoxicity side effects (34).

*Phyllanthus fraternus: P. fraternus* is a tropical plant from family Euphorbiaceae whose pharmaceutical uses have recently attracted pharmacologists’ attention. Aqueous *P*. *fraternus* extract can reduce renal disorders induced by anticancer drugs such as cisplatin and cyclophosphamide. This plant can modulate mitochondrial respiratory disorders and lipids peroxidation (35).

Peach: In Asia, different parts of peach (*Prunus persica)* are used as herbs. Lee et al studied the effect of peach pericarp extract (PPE) on cisplatin-induced nephrotoxicity demonstrated that PPE controlled cisplatin-induced increase in urea and creatinine in mice and modulated nitric oxide, glutathione content, and lipids peroxidation (36). A similar study reported that peach flesh extract had similar effects to peach seeds and also reduced kidney weight loss in animal models (37).

*Sphaeranthus indicus: S. indicus*, which is also referred to as east Indian globe thistle, is used for treating several diseases, but few studies have conducted on this plant. Use of ethanolic extract of* S*. *indicus* 10 days after administration with cisplatin was effective in reducing the nephrotoxicity indices such as BUN level and serum creatinine level. It also increased antioxidant indices (superoxide dismutase, catalase, and glutathione peroxidase) in mice kidney and regulated glutathione level (38).

*Urtica dioica* L.: *U. dioica*, commonly referred to as common nettle, has long been used in traditional medicine. Ozkol et al have studied the antitoxic effects of common nettle in mice kidney and liver after administration with cisplatin. It showed that methanolic common nettle extract exerted preventive effect through decreasing BUN level, lipids peroxidation, myeloperoxidase, and proteins oxidation due to its antioxidant properties. Moreover, it could decrease the activities of certain enzymes such as superoxide dismutase, glutathione S-transferase, catalase, and glutathione peroxidase as well as glutathione content (39).

Soybean: The effect of the extract of soybean* (Glycine max)* on cisplatin-toxified human proximal tubular HK-2 cells was investigated and demonstrated antioxidant effects on H_2_O_2_ (40).

*Chrysanthemum indicum: C. indicum* is an Indian flowering plant. Pongjit et al investigated anti-nephrotoxic effects of this plant and found it can prevent of apoptosis in of human proximal tubular cells and display antioxidant activity against H_2_O_2_ and OH^-^(40).

*Origanum majorana: O. majorana*, commonly called marjoram, is an aromatic plant that can control cisplatin-induced nephrotoxic side effects such as increased renal functional markers including BUN, urea, uric acid, and serum creatinine, and modulate cisplatin-induced oxidative markers (41).

*Ribes diacanthum* Pall: In Mongolia, *R. diacanthum* is used as a remedy for urinary tract complications. Aqueous *R*. *diacanthum* extract in cisplatin-treated mice caused decrease in some toxicity biomarkers such as BUN and serum creatinine, increase in the activities of certain enzymes including heme oxygenase 1, reduction in superoxide dismutase, increase in catalase, and inhibition of lipids peroxidation and other reactive oxygen species (ROS) (42).

*Pueraria tuberosa* DC.: *P. tuberosa* is used in Ayurvedic medicine, India’s primary healthcare system. *P*. *tuberosa* powder prevented cisplatin-induced nephrotoxicity in mice due to antioxidant property. Therefore, this plant can be administered as a supplement to prevent nephrotoxicity (43).

*Glycyrrhizia glabra:* As a crude extract, licorice extract obtained from the dried root of *G. glabra* is widely administered in Chinese traditional medicine. Its nephrotoxic effects are due to antioxidant properties. Administration with licorice extract inhibited all side effects due to cisplatin-induced nephrotoxicity including increased serum creatinine and BUN as well as intensified oxidative stress in mice. However, since the therapeutic efficacy (destruction of tumor cells) of licorice extract was reduced in combination with cisplatin, it is recommended to use this extract alone rather than in combination with cisplatin for exerting anticancer effect (44).

#### 3.1.2. Compounds

*Catechin, epigallocatechin-gallate, and epigallocatechin:* The compounds found in green tea such as catechin, epigallocatechin gallate (EGCG), and epigallocatechin have been reported to be effective in protection against nephrotoxicity (45). A study on cisplatin-treated mice demonstrated that pretreatment with green tea polyohenols (EGCG, and epigallocatechin) decreased toxic changes and cisplatin-induced side effects and improved renal function in the mice under treatment (46). In addition, El-Mowafy et al found that administration of 20-40 mg/kg of green tea catechin and EGCG led to decrease in tumor necrosis factor alpha (TNF-α) and MDA and increased glutathione level. In addition, EGCG can suppress systemic inflammation and prevent oxidative stress and leukocytosis. Therefore, this polyohenol has been reported to prevent incidence of side effects and mortality due to cisplatin-induced nephrotoxicity in rats (47).

Proanthocyanidins: Grape can inhibit apoptotic and necrotic cell processes and prevent anticancer drug-induced tissue toxicity in all cells of the body, due to proanthocyanidins found in grape seed (48). A study indicated that grape seed proanthocyanidins led to decrease in nephrotoxicity induced by drugs, especially anticancer ones. That study showed that grape seed proanthocyanidins could prevent oxidative stress, genomic integrity, and cell death (49). The proanthocyanidins found in grape seed can prevent cisplatin-induced cytotoxic side effects in the kidney through modifying expression of the proteins associated with apoptosis and antioxidant property (50). Gao et al investigated the protective effects of grape seed proanthocyanidin extract on nephrotoxicity in male laboratory mice, and found that cotreatment with proanthocyanidins and cisplatin could result in decreased BUN level, renal index, and serum creatinine. Therefore, proanthocyanidins protects nephrotoxicity through weakening endoplasmic reticulum stress-induced apoptosis via regulating caspase-12 (51). Proanthocyanidins are phenolic compounds whose properties are dose-dependent and that cause improvement of renal function mainly due to antioxidant property (52).

PTY: PTY is an isoflavonoid compound derived from tubers of *Pueraria tuberosa*. Use of PTY increases the activities of catalase and superoxide dismutase, decreases glutathione content and index biomarkers in nephrotoxicity (BUN and serum creatinine). Besides, PTY causes decline in tubular inflammation, damage to DNA, protein cast deposition, and tissue damage. Therefore, the antioxidant properties of PTY can regulate renal function in mice (53).

Resveratrol: Resveratrol is a polyphenol found in white hellebore (*Veratrum grandiflorum* O Loes) root, apple, and grape as well as in other medicinal plants. Resveratrol is a protective agent against chemotherapy drug-induced nephrotoxicity. This compound has antioxidant, anti-inflammatory, and antiapoptotic activities and can suppress cisplatin-induced cytotoxic effects (54-56). Indications of renal injury and toxicity such as increased serum creatinine level and urine proteins in resveratrol-treated mice were reported to be lower than those in controls. Furthermore, administration with resveratrol caused decrease in glutathione depletion and lipids peroxidation (57,58). This compound has anti-inflammatory properties and suppresses oxygen species activity. Resveratrol antioxidant property is associated with decreased 4-HNE and carbonyl adduction (57). A study demonstrated that in cisplatin-treated rats, serum creatinine, urine protein and volume, lipids peroxidation, and glutathione depletion increased. Such changes and nephritis were mitigated in resveratrol-treated rats (59).

Cardamonin: Cardamonin is a flavonoidic compound that is found in various plants including alpinia plant. Study of the nephroprotective properties of alpinia demonstrated that cardamonin led to decrease in caspase-3 expression and Bax/Bcl-2 ratio, and could decrease NOX-1 expression in mice, therefore reduce nephrotoxicity (60).

Ginsenosides: Ginseng contains an active and antioxidant compound named ginsenoside 20(S)-Rg3 that is used as a medicine to treat renal disorders in some regions of the world. Use of this plant-based compound prevents cisplatin-induced apoptosis in porcine LLC-PK1 cells through inhibiting JNK-p53-caspase-3 signaling cascade (61). Furthermore, damage to the LLC-PK1 cells is reduced due to treatment with ginsenosides Rg3, Rg5, and Rk1 and processed ginseng. Moreover, expressions of p53 and c-Jun N-terminal kinase (JNK) proteins that are reduced by cisplatin are restored. This antitoxic activity of ginseng active compounds is due to their anti-inflammatory and antiapoptotic properties (62). Moreover, other compounds of ginseng (Rh4 and Rk3) can decrease the induced nephrotoxicity in the LLC-PK1 cells (63).

Luteolin: Luteolin is a flavonoidic and plant-based compound with anticancer, anti-inflammatory, and antioxidant properties. A study investigated the effect of luteolin demonstrated that cisplatin led to increased levels of bax, caspase-3, and PUMA-α as well as p53 and its phosphorylation activity. Treating mice with luteolin can significantly eliminate side effects and therefore modulate p53-dependent renal tubular apoptosis and consequently nephrotoxicity (64). Moreover, luteolin was reported to inhibit apoptosis of renal cells, and leads to improved renal function and nephrotoxicity indices (histological and biochemical) and prevents accumulation of platinum in mice kidneys through inactivating nuclear factor-kappaB (NF-κB) and decreasing the expression of TNF-α and cyclooxygenase-2 (COX-2) through suppressing oxidative/nitrosative stress by increasing glutathione levels and decreasing 3-nitrotyrosine (3-NT) and 4-hydroxynonenal (4-HNE) formation as well as cytochrome P450 2E1 (CYP2E1) expression (65).

Silymarin: Silymarin is a plant-based flavonoid compound of *Silybum marianum*. Silymarin exerts anti-inflammatory effect through decreasing TNF-α and reduces acute nephrotoxicity through protecting erythrocytes lysis. This plant reduces BUN and serum creatinine and therefore cisplatin-induced nephrotoxicity in patients, which is attributed to its immunomodulatory, antioxidant, and anti-inflammatory properties (66).

Tetrahydrocurcumin: Tetrahydrocurcumin is a compound from curcuminoids of tumeric root that displays anti-inflammatory properties in preventing cisplatin-induced nephrotoxicity through preventing the activities of certain enzymes such as caspase-3 and cyclooxygenase-2 (67).

Lycopene: Carotenoids including lycopene are among the most potent antioxidants and prevent other oxidants from inducing toxic effects in the body. Hassan et al (50) reported that lycopene prevented cisplatin nephrotoxicity in mice through a complex process. Indeed, lycopene exerts its effects through reducing serum creatinine and BUN, preventing increase in MDA and isoprostane, decreasing renal bax protein as well as renal HSP60 and HSP70, increasing Bcl-2 expression, and decreasing lipids peroxidation (68). A study indicated that use of lycopene before or after cisplatin administration could significantly protect the kidney against cisplatin-induced nephrotoxicity and improved blood markers in rats through increasing glomerular filtration and decreasing serum creatinine, urea, and concentration of MDA (69). Erman et al demonstrated that use of lycopene after cisplatin administration caused decrease in BUN and serum creatinine levels and efflux transport proteins MRP2 and MRP4 in the studied animals. Moreover, in the lycopene -treated group, organic cation transporter 1 and 2 proteins and organic anion transport 1 and 3 proteins were regulated. These changes caused reduction in nephrotoxicity (70).

#### 3.1.3. Traditional herbal prescriptions and pharmaceutical formulations

HemoHim: HemoHim is a medicinal plant that is produced by adding the ethanol-insoluble fraction to the total water extract of a mixture of three edible herbs, *Angelica radix*, *Cnidium rhizoma*, and *Paeonia radix*. This drug causes reduction in cisplatin-induced nephrotoxicity through exerting antioxidant properties. A study on mice with cancer under treatment with cisplatin demonstrated that HemoHim could increase the activity of natural killer cells and Tc cells as well as the IL-2 and IFN-gamma secretion from splenocytes. Moreover, in addition to enhancing anticancer effects of cisplatin, HemoHim causes reduction in nephrotoxicity through prevention of tubular cell destruction in the kidneys (71).

Cystone: Cystone is a combination of some plants that is used to treat renal complications in Himalaya and some other regions. Rao et al found that cystone could decrease BUN and serum creatinine of laboratory mice after five days of treatment with cisplatin without disturbing cisplatin function at a specific dose (72). Moreover, in a randomized clinical trial on 49 patients with cancer, treatment with cystone and cisplatin caused significant decrease in levels of BUN, serum creatinine, and serum cystatin C compared to treatment with cisplatin alone. Therefore, cystone can help decrease nephrotoxic side effects of cisplatin without interfering with the treatment process (59).

Wuzhi tablet: Wuzhi tablet is a pharmaceutical formulation derived from ethanolic *Schisandra sphenanthera* extract. The most important chemical compound of this formulation is Schisantherin. Nuclear accumulation of Nrf2 and Nrf2-dependent genes increased in the mice treated with Wuzhi tablet. Actually Wuzhi tablet decreases nephrotoxicity through the activation of Nrf2/ARE pathway in HK-2 cells and the kidney of mice. Therefore Wuzhi tablet can balance defense responses for decreasing nephrotoxicity in mice (73).

### 3.2. Implications and mechanisms

**Figure 1:**
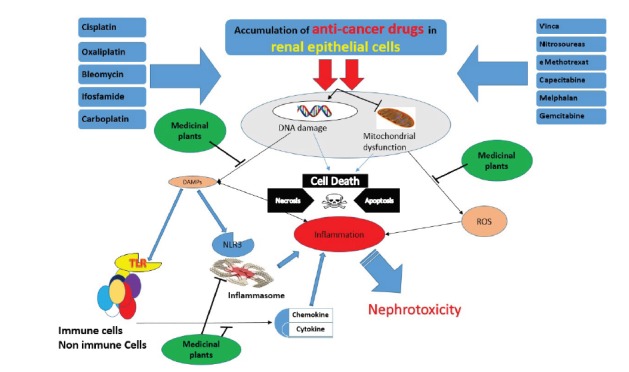
Role of medicinal plants in ameliorating nephrotoxicity-induced by anticancer drugs.

Renal tubular cells are particularly susceptible to toxins. Renal injuries are mainly due to inappropriate exposure of these cells to circulation of chemical compounds and transfer of substances that cause escalation of intracellular toxin concentrations. Overall, chemical compounds or their metabolites exert toxicity through developing covalence or non-covalence bonds in macromolecules or via ability to produce ROS (74). Several mechanisms are likely to induce nephrotoxicity including direct damage to DNA and induction of oxidative stress due to accumulation of drugs in renal cells that can directly or indirectly cause increase in ROS, mitochondrial dysfunction, increase in inflammation factors, renal cell death due to necrosis and apoptosis as well as inflammation (Figure 1). In most cited studies, cisplatin, serving as one of the most important anticancer drugs, was reported to induce nephrotoxicity. This drug enters renal epithelial cells and causes damage to DNA and induction of stress in the endothelial network. Then, the arrangements for apoptosis and necrosis are made through production of ROS and activation of mitochondrial and non-mitochondrial apoptosis-dependent pathways. Besides that, extensive apoptosis and cell death initiate an extensive inflammatory response that may lead to nephrotoxicity and renal damage (2,6).

Moreover, during toxin-induced changes in the renal cells, the proteins and lipids in the membrane, nucleus, lysosome, mitochondria, and cytosol are collectively targeted. In case of oxidative stress, lipids peroxidation and proteins oxidation occur, which exacerbates cell damage. In many cases, mitochondrion is an important target, and adenosine triphosphate (ATP) deficiency leads to cell damage due to aerobic metabolism-dependent renal function. In addition, ATP deficiency causes certain disorders in the process of distributing the genes, which leads ultimately to escalation of calcium ion and cell death. In addition, oxidative stress contributes to cell death through production of cysteine protease (73). Moreover, expansion of renal injuries, particularly acute renal injury, which is due to certain chemical compounds could cause an increase in serum urea and creatinine and decrease in the kidney cleaning capacity. Besides that, decrease in renal perfusion, hematuria, proteinuria, cylinduria, and oliguria are some other side effects of this condition that occur due to inflammation and lysis of the renal cells (75-77). These changes decrease the rate of renal filtration (78). Accordingly, administration with antioxidants could be a novel and proper approach to treat anticancer drug-induced nephrotoxicity (79). Indeed, anticancer drugs exert their destructive effects, and pathways of oxidative stress, inflammatory processes, and cell apoptosis contribute significantly to these changes. The role of antioxidant and anti-inflammatory compounds found in plants and plant-based compounds in removing side effects has been highlighted in most studies (80-84). However, antioxidant compounds and even certain plant-based extracts used to prevent and reduce nephrotoxicity should be administered under supervision a physician and cautiously (85-87). Taken together, given many side effects of anticancer drugs such as nephrotoxicity, bone marrow suppression, and hepatotoxicity, it is necessary to seek out an alternative approach, namely use of medicinal plants or herbal medicines or chemical drugs (88,89). However, there are still some controversies over use of antioxidants alongside anticancer drugs, because it has been demonstrated that excessive use of antioxidants interferes with the process of chemotherapy and cancer treatment (90). Therefore, it seems still essential to determine the active dose and cutoff point of the antioxidant compounds (91-93).

Nephrotoxicity may be affected by certain factors such as drug dosage, use frequency, cumulative dose, aging, gender, smoking, hypoalbuminemia, and history of chronic renal failure that are considered risk factors for cisplatin-induced nephrotoxicity as well. These factors too can be considered confounders and therefore influence the findings (6). Duration of administration with herbal drugs is another issue that may challenge the reliability of the findings. According to this review article and several studies, herbal drugs and their compounds could be used alongside chemical drugs as pretreatment, co-treatment, or post-treatment. Therefore, it can be assumed that increased serum concentrations of active plant-based compounds, chemical drugs, drug interactions, and production of toxic metabolites have been investigated less frequently and most studies have been conducted with animal models. Moreover, given that using serum creatinine and BUN can delay diagnosis of nephrotoxicity, it is necessary to use other biomarkers to diagnose nephrotoxicity and start its treatment earlier. In addition, it is recommended to investigate renal biochemical markers thoroughly as well as to take into account necessary standards to achieve high sensitivity and accelerate study of nephrotoxicity. Determining active dose of the plants and their derivatives to reduce nephrotoxicity should be taken into consideration more seriously.

## 4. Conclusions

According to the current review article, anticancer drugs induce nephrotoxicity especially through activating pathways of oxidative stress, inflammatory processes, and cell apoptosis. The medicinal plants and their derivatives can reduce nephrotoxicity and other side effects induced by these drugs through exerting antioxidant and anti-inflammatory properties (Figure 1).

## Authors’ contribution

SHS, MAS and ASB searched the databases. SHS and MAS wrote the draft. MAS, QY and ASB edited the draft. All authors read and approved the final version.

## Conflict of interest

The authors declared no competing interests.

## Funding/Support

None declared.
